# Supplementary figures and images for: Three-dimensional composition of the photoreceptor cone layers in healthy eyes using adaptive-optics optical coherence tomography (AO-OCT)
Source: PLoS One. 2021 Jan 7;16(1):e0245293. doi: 10.1371/journal.pone.0245293 (PMC7790532; doi:10.1371/journal.pone.0245293)

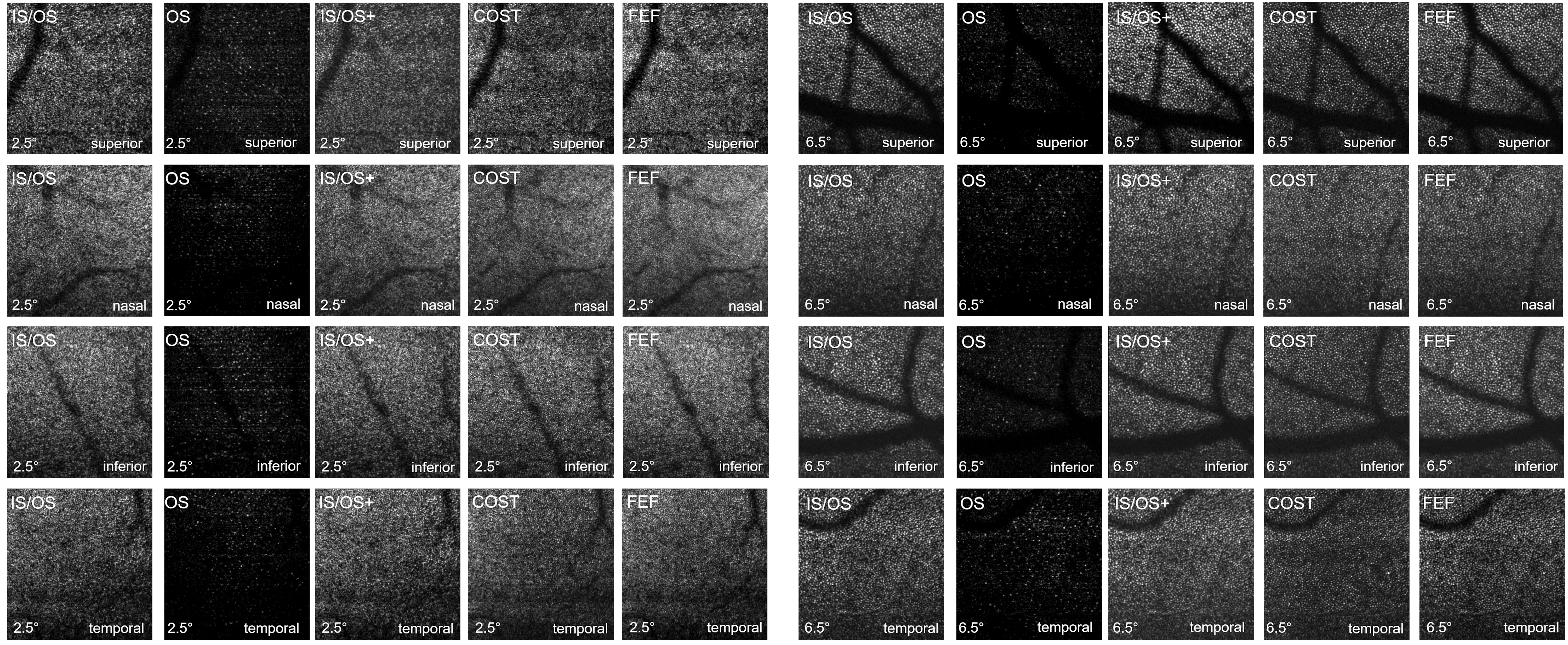

Supplement: S1 Fig — There is no distinctive difference between the separate images of each meridian (compare each of the 4 images within every column). As expected from previous studies, there is a remarkable difference in density and size of the hyper-reflective dots between 2.5° (left half of the image) and 6.5° (right half of the image) foveal eccentricity. (TIF) [file pone.0245293.s001.tif]

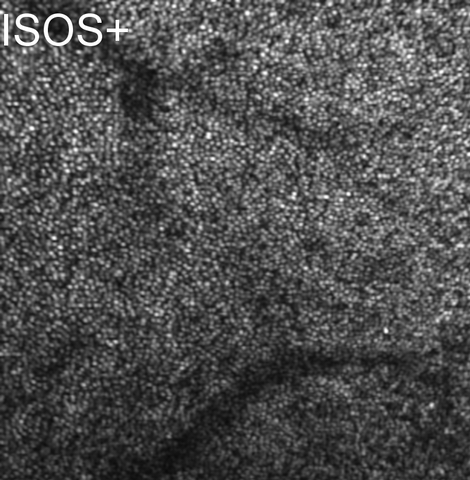

Supplement: S1 File — (GIF) [file pone.0245293.s002.gif]

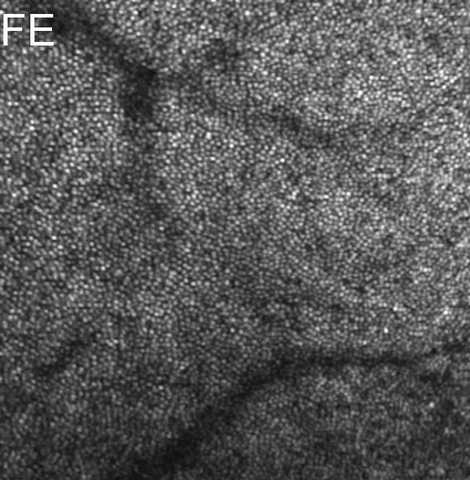

Supplement: S2 File — (GIF) [file pone.0245293.s003.gif]

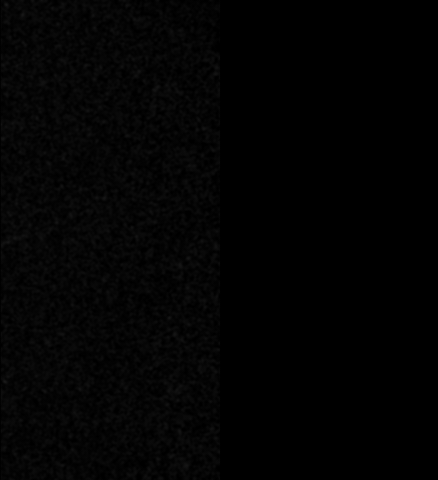

Supplement: S3 File — (GIF) [file pone.0245293.s004.gif]
